# Music-supported motor training after stroke reveals no superiority of synchronization in group therapy

**DOI:** 10.3389/fnhum.2014.00315

**Published:** 2014-05-20

**Authors:** Floris T. Van Vugt, Juliane Ritter, Jens D. Rollnik, Eckart Altenmüller

**Affiliations:** ^1^Institute of Music Physiology and Musicians' Medicine, University of Music, Drama, and Media HanoverHanover, Germany; ^2^Lyon Neuroscience Research Center, CNRS-UMR 5292, INSERM U1028, University Claude Bernard Lyon-1Lyon, France; ^3^BDH-Klinik, Institute for Neurorehabilitational Research (InFo), Teaching Hospital of Hanover Medical SchoolHessisch Oldendorf, Germany

**Keywords:** stroke rehabilitation, music therapy, motor improvement, synchronization, social, shared experience, mood

## Abstract

**Background**: Music-supported therapy has been shown to be an effective tool for rehabilitation of motor deficits after stroke. A unique feature of music performance is that it is inherently social: music can be played together in synchrony.

**Aim**: The present study explored the potential of synchronized music playing during therapy, asking whether synchronized playing could improve fine motor rehabilitation and mood.

**Method**: Twenty-eight patients in neurological early rehabilitation after stroke with no substantial previous musical training were included. Patients learned to play simple finger exercises and familiar children's songs on the piano for 10 sessions of half an hour. Patients first received three individual therapy sessions and then continued in pairs. The patient pairs were divided into two groups. Patients in one group played synchronously (together group) whereas the patients in the other group played one after the other (in-turn group). To assess fine motor skill recovery the patients performed standard clinical tests such as the nine-hole-pegboard test (9HPT) and index finger-tapping speed and regularity, and metronome-paced finger tapping. Patients' mood was established using the Profile of Mood States (POMS).

**Results**: Both groups showed improvements in fine motor control. In metronome-paced finger tapping, patients in both groups improved significantly. Mood tests revealed reductions in depression and fatigue in both groups. During therapy, patients in the in-turn group rated their partner as more sympathetic than the together-group in a visual-analog scale.

**Conclusions**: Our results suggest that music-supported stroke rehabilitation can improve fine motor control and mood not only individually but also in patient pairs. Patients who were playing in turn rather than simultaneously tended to reveal greater improvement in fine motor skill. We speculate that patients in the former group may benefit from the opportunity to learn from observation.

## Introduction

Motor impairments are among the most common and most disabling consequences of stroke (Ward and Cohen, 2004; Dimyan and Cohen, 2011). Since effective therapeutic interventions are scarce (Woldag and Hummelsheim, 2002), several novel interventions including musical activities were developed (Bunketorp Käll et al., 2012; Chong et al., 2014). For example, stroke patients showed significant improvements in fine motor control after music-supported therapy in which they learned to play the piano and drums during several weeks (Schneider et al., 2007; Altenmüller et al., 2009; Amengual et al., 2013; Grau-Sánchez et al., 2013). These beneficial changes persisted in a 3-week follow-up test (Villeneuve and Lamontagne, 2013). The researchers found that patients' musical training transferred to motor benefits in a variety of clinical tasks measuring activities of daily living, revealing gains in fine motor control in these patients. Neurophysiologically, these behavioral improvements were accompanied by auditory-sensory-motor coactivation in the level of the cortex (Rojo et al., 2011) and by a shift in motor excitability patterns of the contra-lesional motor cortex of the patients as assessed with transcranial motor stimulation (Amengual et al., 2013; Grau-Sánchez et al., 2013).

A variety of explanations has been advanced for the performance improvement and the neuroplastic changes due to music-supported therapy in these patients: the brain's use of auditory feedback, the novelty of the intervention, and increased motivation (Altenmüller et al., 2009). However, we wondered whether the fact that music is a social activity played a role in the benefit of music-supported therapy. In healthy populations, music turned out to be an effective tool for supporting pro-social commitment, increasing group cohesion and cooperation (Overy, 2012). The particular aspect of music that is shown to be involved in creating group cohesion is synchronization. Performing (musical) movements in synchrony has been shown to improve feelings of reciprocal likeability (Hove and Risen, 2009), trust (Wiltermuth and Heath, 2009; Launay et al., 2013), pseudo-altruism (Kokal et al., 2011; Valdesolo and Desteno, 2011), and even destructive obedience (Wiltermuth, 2012). As a result of these findings, we hypothesized that music may be a powerful tool for promoting pro-social engagement in a rehabilitation therapy session. In support of this, it has been shown that participants in music therapy rehabilitation are more actively involved and cooperative than in other forms of therapy (Narme et al., 2012).

Furthermore, we hypothesized that greater engagement of patients in their rehabilitation would lead to an improved clinical outcome. Stroke victims often suffer from disturbances in motivation and mood (Caeiro et al., 2013). Social support (Sandin et al., 1994), in turn, has been associated with improved functional outcome after stroke (Glass et al., 1993). Neuroplastic changes of rehabilitation have been proposed to depend on a patients' emotional connection with the activities in question (Sanes and Donoghue, 2000). However, to date, no studies connected these two causalities (synchronization leads to social engagement which leads to functional improvement in motor function). We set out to directly test whether musical synchronization could enhance functional motor outcome after stroke.

The present study aimed to test the potential for music as a tool to create pro-social engagement on the part of patients. In particular, we hypothesized that the aspect of music that might boost social participation is synchronized musical playing. That is, do patients benefit from producing sounds in synchrony? In order to specifically test for the effect of synchronization whilst keeping other factors constant, we divided our patient population into pairs. Some pairs played in synchrony during their therapy whilst others played in turn.

We asked the following questions. First, is playing together in synchrony associated with changes in functional motor outcome? Secondly, we asked whether playing in synchrony or in turn influenced patients' mood. Thirdly, we asked whether patients basic auditory-motor functioning (such as synchronizing to a metronome) was influenced by playing in synchrony or in turn.

## Methods

We assigned patients to one of two groups in a randomized design. Both groups received music therapy in pairs and they played the same selection of finger exercises and children's songs. Patients received 10 therapy sessions of half an hour. The first three therapy sessions were individual and the remaining seven were in pairs. The patients were divided into two groups. Patients in one group played in synchrony (together group) whereas the patients in the other group played one after the other (in-turn group). All patients received therapy in groups of two. Prior to therapy (PRE) and after therapy (POST), all patients completed a battery of tests described below. In between the three individual sessions and the seven joint sessions, we included a short session of measurements (INTER).

### Patient group characteristics

We aimed at obtaining a representative sample of patients from the hospital population. Consequently, we were not able to maintain high homogeneity of patient selection. However, we feel that by making this choice, our results are maximally relevant to clinical practice. Inclusion criteria were:

- light to moderate motor impairment in the upper extremity due to stroke (ischemia or hemorrhage);
- having residual voluntary movement capacity (practically, the patient was required to be able to move the arm and the index finger of the affected hand independently);
- age between 30 and 75 years;
- Barthel Index at least 25;
- right-handedness;
- less than 5 months had passed since patients' stroke at the time of inclusion in the study;
- being able to understand and agree with informed consent to participate.

Exclusion criteria were:

- previous musical training for more than 4 years;
- psychiatric problems (as assessed by standard clinical investigation);
- cognitive impairment or aphasia.

Initially, 36 patients were identified that matched the inclusion criteria and provided informed consent to participate. However, six patients (17%) were released from the hospital prior to finishing our therapy program. Furthermore, two patients (6%) dropped out of therapy because they no longer felt therapy was effective. Our final sample consisted of 28 patients. Patients were assigned quasi-randomly to the groups. Patients were included two or three at a time, since insufficient patients were available to include all patients at the same time. A custom designed computer script was used to quasi-randomly assign patients to groups making sure that (1) the number of patients in the two groups were as close as possible, and (2) the two groups were as closely matched as possible for age, gender, Barthel index, and nine-hole pegboard test score. We report clinical data about these patients in Table 1.

**Table 1:** **Clinical data of the two patient groups**.

	**Together**	**In-turn**	**Statistical comparison**
Number of patients	14	14	
Sex (female/male)	6/8	10/4	Fisher exact test *p* = 0.2519
Age (years)	65.6 (10.5)	67.1 (11.8)	*W* = 85.0, *p* = 0.56
Handedness (R/L)	14/0	13/1	Fisher exact test *p* = 1
Stroke type (Number of patients ischemia/hemorrhage)	12/2	12/2	Fisher exact test *p* = 1
Affected hand (Number of patients R/L)	9/5	8/6	Fisher exact test *p* = 1
Days since stroke (at PRE, days)	40.6 (25.6)	45.6 (29.9)	*W* = 89.5, *p* = 0.71
Lesion site (Number of patients with lesion at that site/Number of patients without lesion at that site)			
Left frontal	2/12	3/11	Fisher exact test *p* = 1
Left temporal	3/11	2/12	Fisher exact test *p* = 1
Left parietal	1/13	0/14	Fisher exact test *p* = 1
Left occipital	0/14	0/14	Fisher exact test *p* = 1
Left subcortical	6/8	7/7	Fisher exact test *p* = 1
Right frontal	2/12	2/12	Fisher exact test *p* = 1
Right temporal	3/11	2/12	Fisher exact test *p* = 1
Right parietal	1/13	1/13	Fisher exact test *p* = 1
Right occipital	3/11	1/13	Fisher exact test *p* = 0.60
Right subcortical	2/12	1/13	Fisher exact test *p* = 1
Barthel index PRE	48.2 (15.0)	48.9 (11.5)	*W* = 96.5, *p* = 0.95
Barthel index POST	72.1 (14.4)	67.7 (14.8)	*W* = 104.0, *p* = 0.54
Faces scale mood rating PRE	2.42 (1.22)	1.85 (1.23)	*W* = 123.5, *p* = 0.22
Therapy duration (days)	18.2 (3.0)	18.4 (5.1)	*W* = 96.0, *p* = 0.94

Continuous data are reported as mean (standard deviation). We report statistical comparison using Fisher exact test whenever appropriate, and Mann–Whitney test otherwise.

### Music training

Patients received 10 sessions of half an hour of piano training over the course of three to four weeks. The day before the first session and a day after the last session were dedicated to individual measurement sessions (PRE and POST), which are described in more details below.

The training program followed the same structure every day. In the beginning of the session, patients played simple finger exercises such as a five-tone scale up and down and other patterns with their paretic hand. Then patients learned to play one from a set of simple children's songs. If patients reached a sufficient level on one of the songs, they would be invited to learn additional songs from the set prepared by the therapist. See Supplementary Materials for more details about the contents of the music-supported therapy.

Each member in the patient pair played on their individual M-Key V2 MIDI controller keyboard that was chosen for its light touch. The two keyboards were connected through the M-Audio Midisport Uno MIDI-to-USB converter to a Linux laptop. The laptop ran a custom made C program that recorded the MIDI events and forwarded them to the software synthesizer Fluidsynth, which generated the sounds using a Steinway sound font. The program additionally changed the MIDI velocity value (loudness) to its maximum value. As a result, all sounds were maximally loud, regardless of how strong patients' keystroke was. This was done to prevent patients' typically very soft keystrokes from being inaudible. The sounds were then played through Creative Inspire T10 speakers (Creative Labs, Inc.) at a comfortable loudness level. The five keyboard keys used in the therapy were numbered. Songs and exercises were then written in a simplified musical notation as numbers in tabular form and presented visually to the patients as a memory aid (see Supplementary Materials for more information).

Patients played the piano with the hand of their affected extremity only. The therapist stood next to the patient and supported the patient's arm when so required. The patients were always encouraged to make as many of the movements by themselves as possible. For those patients who were more severely affected, the therapist initially pointed to the fingers or moved them gently, encouraging the patient to make the movements unassisted on the next trial. Throughout therapy, the therapist's aim was always to allow the patient to function as independently as possible instead of becoming dependent on the therapist.

In the together-condition, the two patients played different voices of the same musical materials (finger exercises or songs) in synchrony. The therapist indicated the tempo and started the patients at the same time. By contrast, in the in-turn group, patients always played one after the other and never in synchrony. While one patient was playing, the other patient waited.

### Nine-hole pegboard test

The nine-hole pegboard test (9HPT) is a clinical test to assess fine motor control. The patients' task is to place nine small sticks one by one (pegs) in nine holes and take them out again (Mathiowetz et al., 1985; Parker et al., 1986; Heller et al., 1987). The patients were seated comfortably with their affected arm resting on the table. The 9HPT board is placed within easy reach of the patient, with the side with the peg container at the side of the tested arm. The experimenter held a stopwatch that was started once the patient touched the first peg, and stopped once the patient released the last peg. This test was performed during the PRE and POST measurement sessions.

### Finger tapping measurements

We investigated patient's finger tapping performance of the affected hand as a measure of fine motor control. Three different tapping conditions were measured: (1) paced thumb-to-index tapping, (2) index finger speed tapping, (3) middle finger speed tapping. The tests are described in detail in what follows. In all conditions, patients were seated comfortably at a table on which they rested their arm. In order to have a portable, flexible and yet maximally accurate measurement of finger tapping performance, we custom-designed a measurement device. Finger motion was recorded by a triaxial accelerometer (ADXL 335) attached gently to the patient's index or middle finger tip (depending on the task). Tap contact was measured by a force sensitive resistor (FSR SEN09375), which consisted of a small sheet whose electrical resistance changes upon contact in a way that depends on the contact force. Both sensors were read out by an Arduino Duemilanove experimentation board running a custom made C program to sample sensors at 3 kHz. The data was then transferred online over USB to a Linux laptop running a custom Python program allowing the therapist to preview the data. We made the blueprints of the device set up as well as the custom programs available online for free for future research groups to use (http://github.com/florisvanvugt/immmotion).

In paced thumb-to-index tapping, patients were instructed to tap as regularly as possible in time with a metronome at 69 BPM (i.e., 1.15 Hz) during 60 s from the first tap (Calautti et al., 2006). The metronome sound was generated using direct digital synthesis (DDS) by the Arduino experimental board as follows. Essentially, we created a wave table (440 Hz, 20 ms) which was written to a PWM pin connected to an audio jack plug. A set of Creative Inspire T10 loudspeakers (Creative Labs, Inc.) were plugged into this connector. The patient was instructed to tap as follows. The side of the hand (at the little finger) rested lightly on the table and the fingers were held in a relaxed posture (neither at maximum flexion nor maximal extension). The index finger and thumb moved to touch each other and then moved apart again to a distance of about 5 cm (but at least 2 cm). The thumb-to-index tapping movement was chosen because it was previously argued to be more natural and a more reliable reflection of activities of daily living (Okuno et al., 2006).

In index finger speed tapping, we measured the maximum tapping rate and variability during approximately 14 s (measured from the first finger tap). Patients rested their elbow on the table and the patients' hand was palm down on the table. The fingers were held in a relaxed posture close to maximal extension but slightly bent so that the position could be sustained without muscular effort. No metronome was used in these speed tapping trials. The patients were instructed to tap as fast and as regularly as possible, lifting their finger at least 2 cm above the table on each cycle. The force sensor surface was placed on the table and the patients were instructed to tap on the same spot every time. In middle finger speed tapping, the procedure was the same as with index finger speed tapping but switching to the middle finger.

The raw data files containing the force trace over time were preprocessed using a custom developed python script (we do not report the accelerometer data here). The script discarded the first and last 0.5 s of data from the recordings and then converted the force sensor trace into Newtons using a previously established calibration table. We then smoothed the signal using a 160-sample Bartlett window (which amounted to approximately 53 ms at our sample rate). The script detected the tap onset landmarks (a sudden impact) when the force exceeded 0.05 Newton. Tap offsets (a release of contact from the tapping measurement surface) were defined as the time point when the force trace dropped below 0.05 Newton again at least 40 ms after the last tap onset. Similarly, the next onset was restricted to occur at least 75 ms after the last tap offset. All data files with their landmarks were furthermore visually inspected to ensure our method of analysis did not introduce any artifacts. In a number of cases the 0.05 Newton onset/offset threshold was adjusted manually to compensate for the fact that some patients tapped too softly or off the sensor surface. We furthermore recorded the maximal tapping force between subsequent tap onset and tap offsets; the intervals between adjacent onsets, which will be referred to as the inter-tap-intervals (ITIs) in what follows; and the duration between the tap onset and tap offset (tap dwell phase duration). Next, we discarded the ITIs that were larger than 2000 ms since these reflected pauses or interruptions in the patient's tapping behavior (such as asking the experimenter whether to continue tapping) instead of the patient's motor capacity. We also discarded ITIs shorter than 120 ms since there were disproportionately many as a result of double-tap recordings.

### Mood test: Profile of mood states

Patients' mood was established using the Profile of Mood States (POMS) (Lorr et al., 1971). The short form has 35 adjectives (items). For each adjective the patients rated to what extent they are applicable to their mood over the last week, on a scale from 1 (not at all) to 5 (very strongly). The items load onto four category sub-scores: depression/anxiety, fatigue, vigor, and hostility (Curran et al., 1995). We used a previously validated German translation (Bullinger et al., 1990). The questionnaire was administered at PRE and POST. The experimenter read each of the items to the patients who then responded verbally.

### Mood test: Faces scale

In order to obtain a quick estimate of the development of a patient's mood throughout the therapy, we used a mood scale of faces (Kunin, 1955; Andrews and Crandall, 1976; McDowell, 2006). Patients were presented a list of smiley faces ranging from very happy to very sad (see Supplementary Materials). Patients were asked at the PRE measurement session which face best represented how they were feeling by pointing to the corresponding face. At the beginning of each therapy session, patients were asked to point how they had felt since the previous session. At the end of each therapy session, too, patients were asked again how they had felt during the therapy session. At the end of each joint therapy session, the patients were asked individually how they felt about the partner patient with whom they received therapy. The patients pointed to one of the faces in such a way that this was not seen by their partner so as to avoid social pressure effects. Finally, during the POST measurement patients were again asked to select the face representing how they felt at present. The therapist wrote down the letter code corresponding to the chosen face without allowing either patient to see the letter in question.

### Ethics

This study was performed in accordance with ethical guidelines proposed by the Medical University Hanover (MHH). The protocol was approved by the ethics board on 20 April 2011 (nr. 1056-2011).

### Data analysis

We performed parametric ANOVA whenever the data quantity and distribution could reasonably be assumed to fulfill its assumptions. We detected deviations from sphericity using Mauchley's Test and whenever it was significant we applied the Greenhause-Geisser correction. In those cases, we indicated significance as p_GG_ and omitted the uncorrected *p*-value for the sake of brevity. We report generalized effect sizes η^2^_G_ (Bakeman, 2005). Groups were then compared using Tukey HSD contrasts.

## Results

### Nine-hole pegboard test

**Figure 1:**
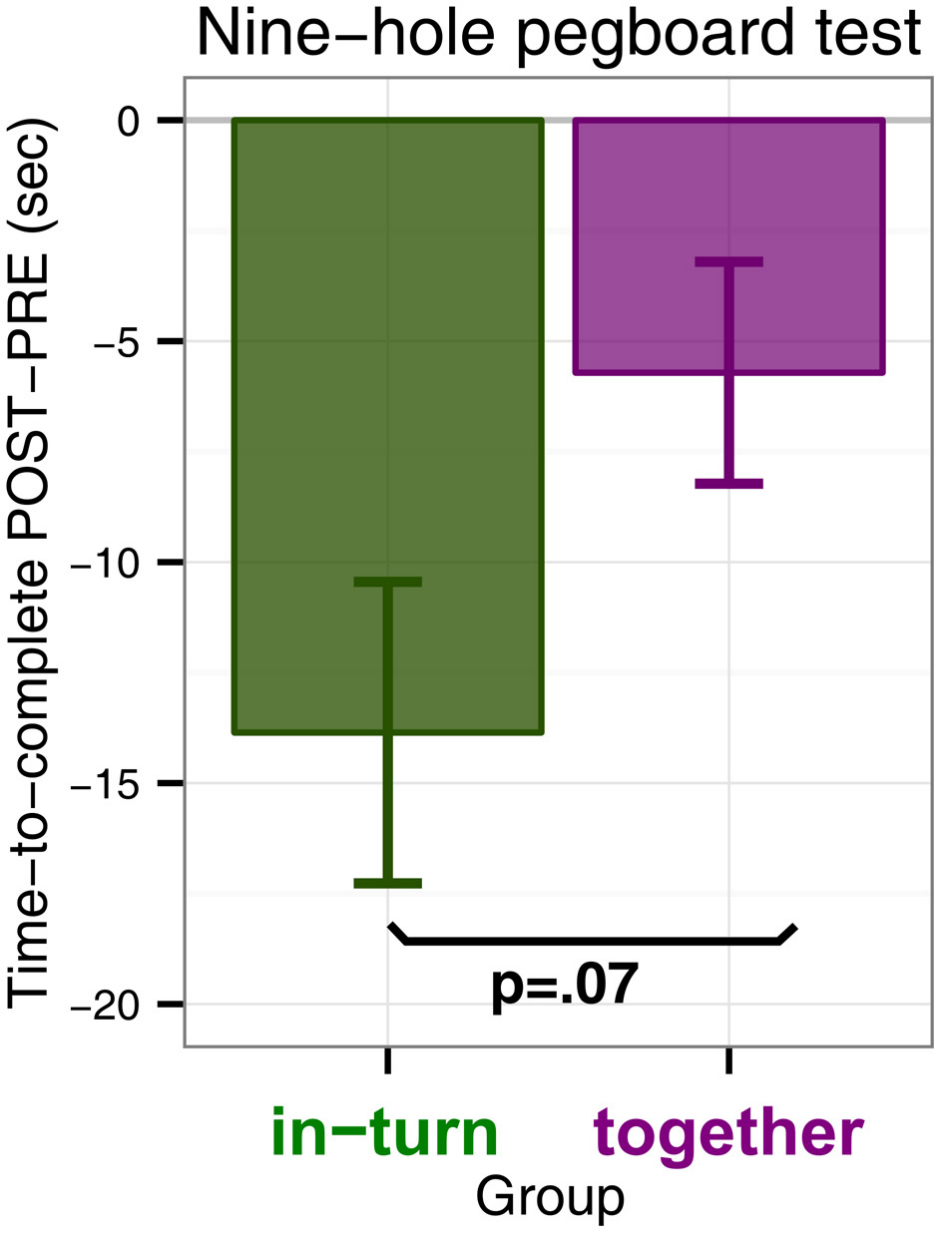
**Nine-hole pegboard test scores measured as the difference in time-to-complete POST minus time-to-complete at PRE (in seconds)**. A negative difference indicates that patients completed the Nine-hole pegboard test faster after therapy (POST) than before (PRE).

We performed an ANOVA with time to complete the pegboard test as dependent variable and factors group (in-turn or together) and measurement (PRE or POST). There was a main effect of time point [*F*_(1,26)_ = 21.35, *p* < 0.0001, η^2^_G_ = 0.02] which indicated that both groups performed the 9HPT faster after therapy than before. Furthermore, there was a trend for an interaction effect between group and time point indicating that in-turn group tended to improve more than the together group [*F*_(1,26)_ = 0.70, *p* = 0.065, η^2^_G_ = 0.004] (Figure 1). There was no main effect of group [*F*_(1,26)_ = 0.74, *p* = 0.40]. We feel that some caution may be needed in interpreting the interaction trend, since the in-turn group performed the test slightly slower at the PRE measurement (*M* = 72.4 s, *SD* = 32.8 s) than the together group (*M* = 42.5 s, *SD* = 36.8 s). However, this difference was not significant [*t*_(26.7)_ = 1.13, *p* = 0.27]. Furthermore, the effect might reflect two patients with larger improvement scores. However, these patients were not more than 3 SD away from the overall mean improvement or the mean improvement per group and were therefore not discarded as outliers.

### Finger tapping tests

#### Index finger unpaced tapping

The PRE measurement of one patient (in the in-turn group) was invalid due to technical reasons and this patient was therefore removed from further analysis. We pooled the taps that were recorded before and after each therapy session and then computed the tapping speed and variability as follows. Speed was calculated as the median of the intervals (in ms). Variability was calculated by first discarding the taps that were 3 SD longer or shorter than the mean for that block, taking the standard deviation of the remaining intervals and then divided it by the mean for that block to obtain the coefficient-of-variation (CV in percent). We found no initial differences in tapping speed between the groups [*t*_(18.8)_ = 1.09, *p* = 0.29] or in tapping variability [*t*_(24.8)_ = −1.2, *p* = 0.24].

**Figure 2:**
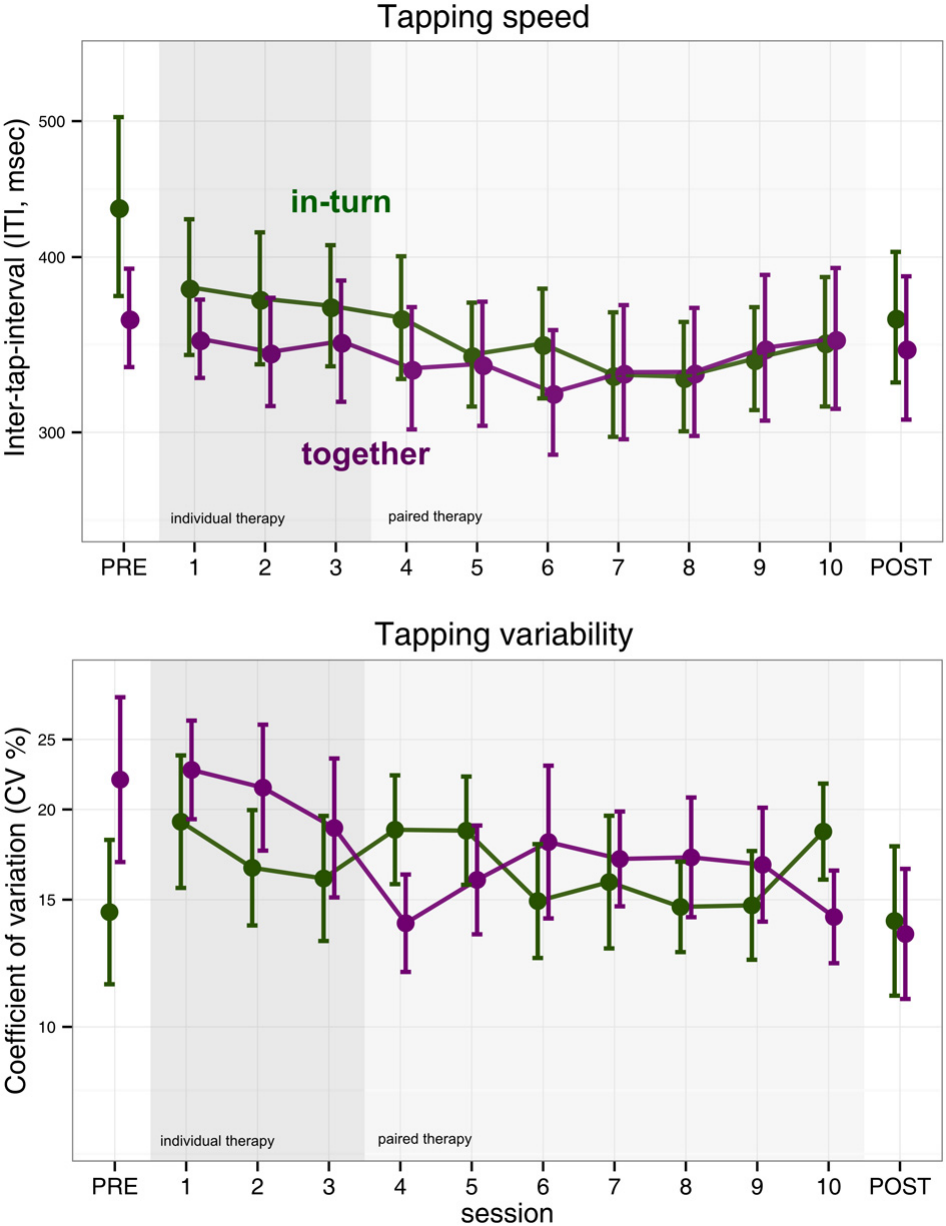
**Index finger tapping speed and regularity**. Error bars indicate the standard error of the mean.

We performed an ANOVA on the log-transformed median tapping interval with factors session (PRE, POST, and the 10 therapy sessions) and group (in-turn, together). The main effect of group was not significant [*F*_(1,25)_ = 0.15, *p* = 0.70]. However, the main effect of measurement session was significant but became only a statistical trend after sphericity corrections [*F*_(11,275)_ = 2.20, *p* = 0.01, *p*_GG_ = 0.10, η^2^_G_ = 0.02]. There was no interaction between group and session [*F*_(11,275)_ = 0.72, *p* = 0.72, *p*_GG_ = 0.53] (Figure 2).

We performed the same ANOVA with log-transformed coefficient-of-variability (CV) as dependent measure. We found no effect of group [*F*_(1,25)_ = 0.09, *p* = 0.76], measurement session [*F*_(11,275)_ = 1.43, *p* = 0.16, *p*_GG_ = 0.20] and no interaction [*F*_(11,275)_ = 1.51, *p* = 0.12, *p*_GG_ = 0.17] (Figure 2).

#### Middle finger unpaced tapping

Middle finger tapping was measured before (PRE) and after (POST) therapy. There were no differences in initial tapping speed [*t*_(13.0)_ = 1.76, *p* = 0.11]. There was a statistical trend for the in-turn group to tap more regularly (*M* = 17.2, *SD* = 9.9% CV) at the PRE measurement than the together group (*M* = 30.2, *SD* = 19.0% CV) [*t*_(24.0)_ = −1.77, *p* = 0.09].

An ANOVA with factors group (in-turn, together) and measurement session (PRE, POST) revealed no effect of group on middle finger tapping speed [*F*_(1,24)_ = 1.89, *p* = 0.18]. There was no effect of measurement session [*F*_(1,24)_ = 1.86, *p* = 0.18] and no interaction [*F*_(1,24)_ = 0.20, *p* = 0.66].

The same ANOVA was performed with tapping variability as dependent variable. We found no effect of group [*F*_(1,24)_ = 0.88, *p* = 0.36] or recording session (PRE, POST) [*F*_(1,24)_ = 0.85, *p* = 0.36]. There was a statistical trend for an interaction between group and session [*F*_(1,24)_ = 3.29, *p* = 0.08, η^2^_G_ = 0.04]. However, in light of the subtle differences in middle finger tapping variability at the PRE measurement, we interpreted these findings as regression toward the mean.

#### Index-to-thumb paced tapping

**Figure 3:**
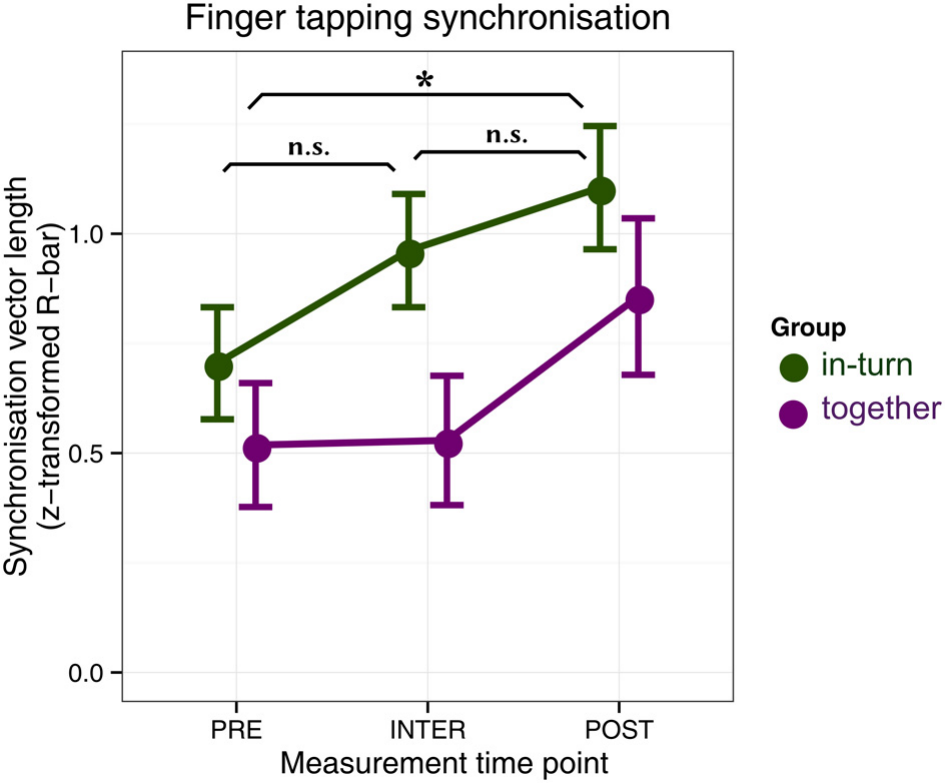
**Synchronization tapping performance before therapy (PRE), in between the individual and joint sessions (INTER), and after therapy (POST)**. Error bars indicate standard error of the mean. Significance of the main effect (across groups) is indicated: ^*^p < 0.05.

Two patients were eliminated from further analysis because during one session their tapping was too soft to be reliably assessed (both from the *together* group). We used circular statistics to quantify the time-lock (synchronization) between patients' finger tap onsets and the metronome click onsets (Fisher, 1995). We then performed a repeated-measures ANOVA with factors group (together, in-turn) and measurement time point (PRE, INTER, and POST) (Figure 3). The main effect of group was not significant [*F*_(1,24)_ = 2.54, *p* = 0.12]. There was a main effect of recording time-point [*F*_(2,48)_ = 10.98, *p*_GG_ = 0.0001, η^2^_*G*_ = 0.09]. This effect reflected the fact that patients' tapping was more synchronized after therapy relative to before (*p* = 0.036). There were no differences between the PRE and INTER measurements (*p* = 0.60) or the POST and INTER measurements (*p* = 0.27). There was no interaction between group and time-point [*F*_(2,48)_ = 1.28, *p*_GG_ = 0.29].

### Mood tests

**Figure 4:**
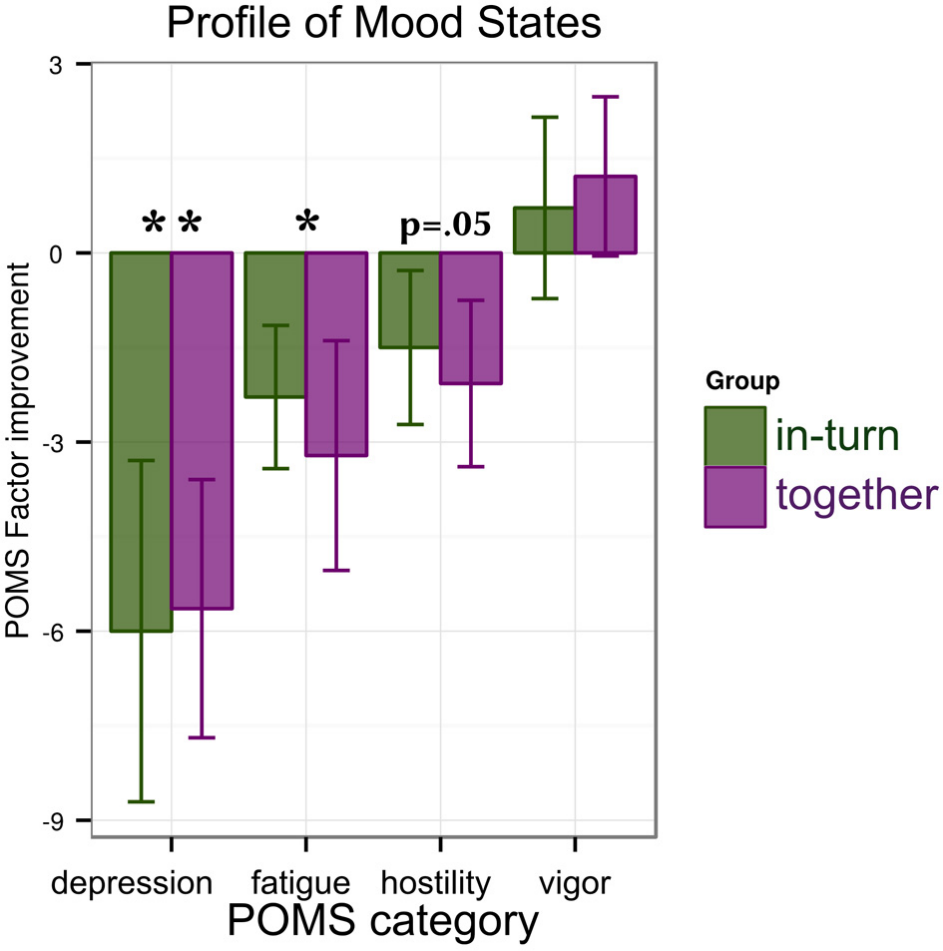
**Results of the mood tests: patients show reductions in depression/anxiety and fatigue that are similar between groups**. A trend for improvement (decrease) in hostility is observed. Significance is indicated as follows: ^*^p < 0.05, ^**^p < 0.01.

For each factor of the POMS (depression/anxiety, fatigue, hostility, and vigor) we performed an ANOVA with factors group (together or in-turn) and time point (PRE, POST). We found a main effect of time point reflecting a reduction in depression [*F*_(1,26)_ = 11.76, *p* = 0.002, η^2^_G_ = 0.09] and fatigue [*F*_(1,26)_ = 6.56, *p* = 0.02, η^2^_G_ = 0.07]. No change was found in vigor [*F*_(1,26)_ = 1.01, *p* = 0.32] and a trend for improvement in hostility [*F*_(1,26)_ = 3.95, *p* = 0.06]. There were no main effects of group [all *F*_(1,26)_ < 0.45, p > 0.51] or interactions between group and time point [all *F*_(1,26)_ < 0.19, *p* > 0.67] (Figure 4).

### Faces scale mood ratings

Patients were asked to rate their own mood on the faces scale, both during the PRE and POST measurement sessions and during the therapy sessions. There were no differences in rating between the groups at the PRE measurement [Mann-Whitney U, *Z* = −1.22, *p* = 0.22]. The in-turn group patients' self-mood ratings improved during therapy [Friedman test χ^2^(11) = 27.36, *p* = 0.004] as did those of the together group [Friedman test χ^2^(11) = 36.08, *p* = 0.0001]. There were no differences in rating during the POST measurement session [Mann-Whitney U, *Z* = −0.62, *p* = 0.54].

Patients were furthermore invited to rate how they experienced the therapy sessions. There was a tendency for the in-turn group to rate the first (individual) session more positive than the together group [Mann–Whitney U, *Z* = −1.76, *p* = 0.08], although first they still received therapy individually. This difference had disappeared by the third session [Mann–Whitney U, *Z* = 0, *p* = 1.00]. During the paired therapy (sessions 4–10), the in-turn group became more positive as therapy progressed [Friedman χ^2^(6) = 13.87, *p* = 0.03] but the together group stayed at the same level [Friedman χ^2^(6) = 7.56, *p* = 0.27]. There were nevertheless no differences in rating between the groups at the last (tenth) session [Mann–Whitney U, *Z* = −0.25, *p* = 0.80].

**Figure 5:**
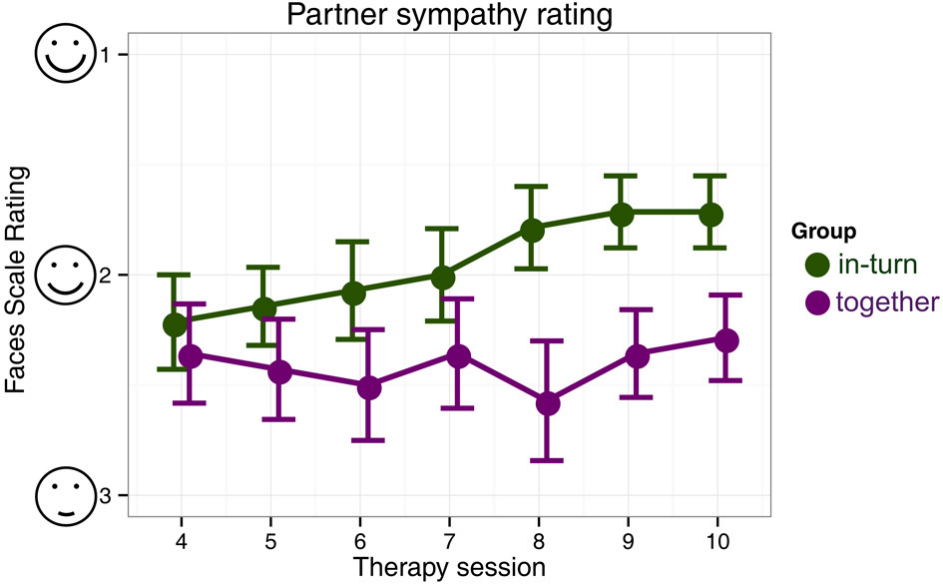
**Patient's likeability rating of their therapy partner**. We indicate mean and standard errors of the ratings for each group for clarity of presentation, although our statistical analysis was non-parametric.

In the partner sympathy ratings, there were no differences in rating between the two groups in the first paired session (session 4) [Mann–Whitney U, *Z* = −0.55, *p* = 0.58]. The in-turn group showed a marked improvement in their rating of their therapy partner [Friedman χ^2^(6) = 25.12, *p* = 0.0003] whereas the together group showed no change in rating [Friedman χ^2^(6) = 4.98, *p* = 0.55] (Figure 5).

## Discussion

Our study was the first to implement music-supported therapy with pairs of patients instead of providing therapy to patients individually. We hypothesized that playing in synchrony would improve patient's social engagement and, through this greater engagement, improve their motor outcome. We controlled for potential benefits of patients sharing their musical rehabilitation experience (Overy, 2012) by having all patients receive therapy in pairs.

Firstly, our results reveal that music-supported stroke rehabilitation can be effective not only when patients are treated individually (as in previous studies) but also in pairs. We found improvements in patients' fine motor control in the 9HPT and synchronization tapping. The finding that music-supported rehabilitation is effective in pairs has practical implications. Paired therapy could considerably reduce the time investment on the part of the therapists. Furthermore, patients showed reductions in depression and fatigue. This indicates that music may have a beneficial effect on mood, in line with previous findings in healthy participants (Seinfeld et al., 2013).

Surprisingly, we found no clear improvements in index or middle finger tapping. This is in contrast to previous studies of music-supported therapy that reported improvements in finger tapping frequency (Schneider et al., 2007; Rodriguez-Fornells et al., 2012; Amengual et al., 2013; Chong et al., 2014) as a result of music-supported therapy. In the case of finger tapping speed, the absence of overall improvement could be due to the fact that finger tapping speed tended to show a u-shaped curve (Figure 2). Patients appeared to improve finger tapping speed in the first half of the therapy but then showed a tendency for a rebound in the second half of the therapy. Alternatively, there may be an effect of which therapist implements music-supported therapy on the rehabilitation outcome. On the other hand, we did find clear improvements in synchronization tapping.

The 9HPT showed a difference in rehabilitation outcome between patients playing in synchrony and patients playing in turn. Contrary to our hypothesis (that patients in the together group would show the greatest benefit), in this test we found a statistical trend for patients in the in-turn group to benefit more.

How could one explain that patients playing in-turn would show greater benefit than patients playing synchronously? We speculate that patients in the in-turn group may benefit from the opportunity to learn through observation. In healthy participants, seeing others perform a motor task leads to motor facilitation (Ménoret et al., 2013) and motor learning (McCullagh et al., 1989; Hodges et al., 2007; Wulf and Mornell, 2008) on the part of the observer. In particular, observers appear to benefit from observing both experts and novices perform a motor task, thus learning from errors as well as exemplary performance (Andrieux and Proteau, 2013). As a result, action observation has been proposed recently as a tool for motor-rehabilitation after stroke (Garrison et al., 2010, 2013; Sale and Franceschini, 2012). This finding suggests that stroke patients undergoing rehabilitation may benefit from first observing a therapist perform movements and then a patient peer perform those same movements, as they did in our in-turn group. In this way, observation during music-supported therapy might improve patients' rehabilitation outcome.

An alternative explanation for our findings is that the simultaneously occurring sounds in the together-condition confused patients, preventing them from dissociating sounds that they self-generated from those that were generated by their partner. This could have prevented the motor system from learning from auditory feedback (Altenmüller et al., 2009). A future study could remedy this problem by providing the two patients in each pair separate headphones in which their own sounds are louder than those of their partner. Another alternative explanation is that patients in the together-group were overwhelmed by the higher task demands. In this group, patients were required not only to produce the correct sequence of keystrokes, but also at the same time as their partner. This required them to observe the other person or listen to their keystrokes and predict when the next keystroke was going to occur (Keller and Repp, 2008; Sebanz and Knoblich, 2009; Pecenka and Keller, 2011). One can argue that coordinating one's actions with that of another person's actions is more challenging than performing the same actions alone. It is possible that the task demands in the together group were too high for the patients, causing the patients to be overtaxed and distracted or confused. This would provide an alternative explanation of the trend finding that the in-turn group shows greater rehabilitation benefit than the together group.

Furthermore, results indicate that patients in the in-turn group grew to like each other more over the course of therapy. This is contrary to previous findings where people moving in synchrony liked each other more than people who did not (Hove and Risen, 2009). Perhaps this difference between our study and previous ones is due to auditory-motor malfunctions in stroke patients, in line with previous suggestions (Rodriguez-Fornells et al., 2012). We found no differences in finger synchronization tapping performance between the groups, suggesting that the overall improvement in synchronization was due to a general improvement in motor capacity and not the fact that one group trained to synchronize during therapy. Similarly, the task of synchronization to another person may be so demanding for patients that the mechanisms that usually mediate synchrony-induced social effects (Wiltermuth and Heath, 2009; Wiltermuth, 2012) were unavailable.

At the outset of this study we had hypothesized two causalities. First, playing in synchrony would increase social engagement on the part of the patients. Second, this greater social engagement would then increase motor rehabilitation outcome. We found no evidence for the first causality. Instead, patients performing in turn rated their partner higher in sympathy ratings. As for the second causality, groups performed mostly similar with perhaps a small advantage for the group playing in turn. This suggests that greater social engagement might indeed improve motor outcome, in line with previous studies.

A limitation of this study is that we have not tested a control group who did not receive any musical intervention. As a result, effects found here that do not differ between groups cannot strictly be attributed to the musical intervention. However, the advantage of this approach is that any differences between the groups are likely due to the principal experimental manipulation (playing together vs. playing in turn). Our patient sample was relatively small and heterogeneous and the exact lesion sites of the stroke were unknown to us. Future studies could correlate lesion localization maps to performance and functional motor outcome of patients undergoing music-supported therapy in order to establish which patient groups might benefit maximally from music-supported therapy.

### Conflict of interest statement

The authors declare that the research was conducted in the absence of any commercial or financial relationships that could be construed as a potential conflict of interest.
