# Supplementary material for: Music-supported motor training after stroke reveals no superiority of synchronization in group therapy
Source: Front Hum Neurosci. 2014 May 20;8:315. doi: 10.3389/fnhum.2014.00315 (PMC4033001; doi:10.3389/fnhum.2014.00315)

Supplementary Materials to **Music-supported motor training after stroke reveals no superiority of synchronisation in group therapy**

Order of the measurements

Below is the order in which the measurements were performed at each of the sessions. This order was not randomised but kept the same since we aimed to investigate group differences and not test differences between tests. Details about the measurements are found in the main manuscript file.

PRE (dedicated measurement session before therapy)

- Briefing about the study
- Patient consent
- Faces scale
- Nine hole pegboard test
- Finger tapping:
  - Paced index-to-thumb tapping during one minute
  - Index finger speed tapping for 15 seconds
  - Middle finger speed tapping for 15 seconds
- Profile of Mood States
- Social questionnaire

BEGIN (at the beginning of each single therapy session)

- Faces scale to rate the own mood
- Finger tapping: index finger speed tapping

END (at the end of each single therapy session)

- Faces scale to rate the own mood
- Faces scale to rate the sympathy for the partner (from the fourth session onwards)
- Finger tapping: index finger speed tapping

INTER (dedicated measurement session after the therapy)

- Finger tapping: Paced index-to-thumb during one minute

POST (dedicated measurement session after the therapy)

- Faces scale
- Nine Hole Pegboard Test
- Finger tapping:
  - Paced index-to-thumb during one minute
  - Index finger speed tapping for 15 seconds
  - Middle finger speed tapping for 15 seconds
- Profile of Mood States
- Social questionnaire

All patients were divided into two groups, the in-turn group and the together group. After 3 sessions of individual training the patients played after another (in-turn group) or together (together group).

Faces Scale

We used the faces scale to obtain an estimate of the patient's mood quickly. The patient pointed to one of the faces and the experimenter noted the corresponding letter.

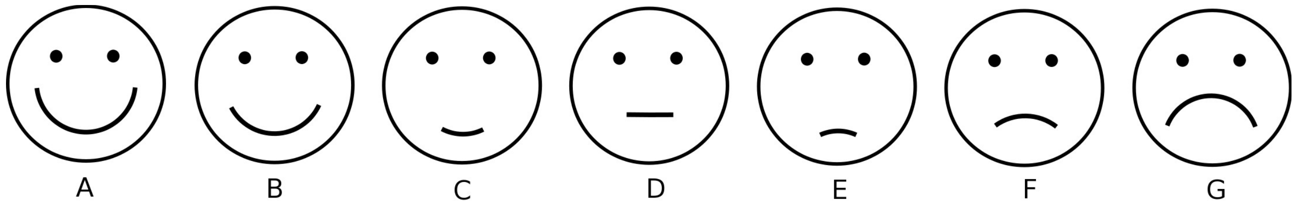

The figure above indicates the letter code that is given for each face, but on the sheet that the patient sees, these letters are absent so as to ensure he or she cannot simply recall the letter.

Therapy program

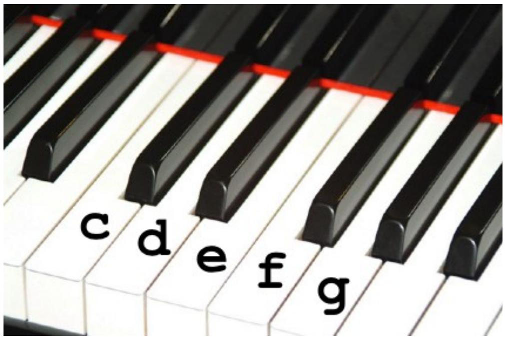

- The patient plays with the paretic arm/hand.
  - right hand c3-g3
  - left hand c4-g4
- The patient should play in a comfortable position. This is why the position of the keyboard should fit to the natural bearing of the patient.
- The tempo of the exercises should be slowly (60 bpm)
- The therapist should support all of the patients' movements but should look to it that the patients try to play alone.

- **Musical materials**

The exercises are all played during a therapy session. The therapist ensures not to judge patients' performance as „correct" or „incorrect". Each session starts with the first exercise and continues with the other exercises always in the same order.

**Exercise 1a**	**Run:**	**c d e f g**	**g f e d c**	(~5x)
**Exercise 1b**	**Repeated run:**	**c c d d e e f f g g**	**g g f f e e d d c c**	(~5x)
**Exercise 2a**	**Jump:**	**c g c**		
**Exercise 2b**	**Repeated jump:**	**c c g g c c**		
**Exercise 3a**	**Mix:**	**c e g**	**f d c**	
**Exercise 3b**	**Repeated mix:**	**c c e e g g**	**f f d d c c**	

Simple children's songs (with numeric presentation)*
„*Hänschen klein*"
„*Freude schöner Götterfunken*"
„*Der Kuckuck und der Esel*"

Fingers were coded numerically

- right hand
  c' = 1 = thumb
  d' = 2 = index finger
  e' = 3 = middle finger
  etc.

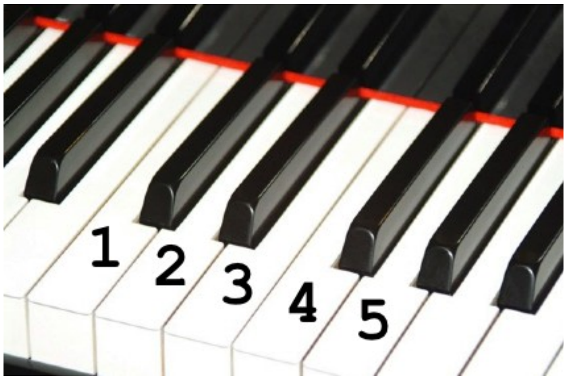

- left hand
  c' = 5 = little finger
  d' = 4 = ring finger
  e' = 3 = middle finger
  etc.

Right Hand

Exercise 1

1 2 3 4 5

5 4 3 2 1

Exercise 2

1 5 1

Exercise 3

1 3 5

4 2 1

„Hänschen Klein“

5 3 3

4 2 2

1 2 3 4 5 5 5

5 3 3

4	2	2					
1	3	5	5	1			
2	2	2	2	2	3	4	
3	3	3	3	3	4	5	
5	3	3					
4	2	2					
1	3	5	5	1			

„Der Kuckuck und der Esel“

5	3	5	3	5	4	4	
4	2	4	2	4	3		
5	4	3	3	3	3	4	4
4	3	2	2	2	2	3	3
1	1	2	3	4	5		
4	3	3	2	2	1		

„Freude schöner Götterfunken“

3	3	4	5

**5**	**4**	**3**	**2**			
**1**	**1**	**2**	**3**	**3**	**2**	**2**
**3**	**3**	**4**	**5**			
**5**	**4**	**3**	**2**			
**1**	**1**	**2**	**3**	**2**	**1**	**1**

Left hand

Exercise 1

**5**	**4**	**3**	**2**	**1**
**1**	**2**	**3**	**4**	**5**

Exercise 2

**5**	**1**	**5**

Exercise 3

**5**	**3**	**1**
**2**	**4**	**5**

„Hänschen Klein“

1 3 3

2 4 4

5 4 3 2 1 1 1

1 3 3

2 4 4

5 3 1 1 5

4 4 4 4 4 3 2

3 3 3 3 3 2 1

1 3 3

2 4 4

5 3 1 1 5

„Der Kuckuck und der Esel“

1 3 1 3 1 2 2

2 4 2 4 2 3

1 2 3 3 3 3 2 2

2 3 4 4 4 4 3 3

5 5 4 3 2 1

2	3	3	4	4	5

„Freude schöner Götterfunken“

3	3	2	1			
1	2	3	4			
5	5	4	3	3	4	4
3	3	2	1			
1	2	3	4			
5	5	4	3	4	5	5

The patients in the together group played all exercises at first synchronously. After a while they played different exercises in combination. For example one patient played the first part of exercise one whereas the other patient played the second part. After a while of practicing we combined the song “Hänschen Klein” (melody) with exercise two (bass line).

Finger tapping measurements

Below is an example of the captured signal that registers two taps (as part of a pilot measurement study). As you can see, we use the smoothed signal to detect the onset and offset of the taps. We furthermore detect the maximum force between the tap onset and offset, as well as the corresponding time value tMaxForce.

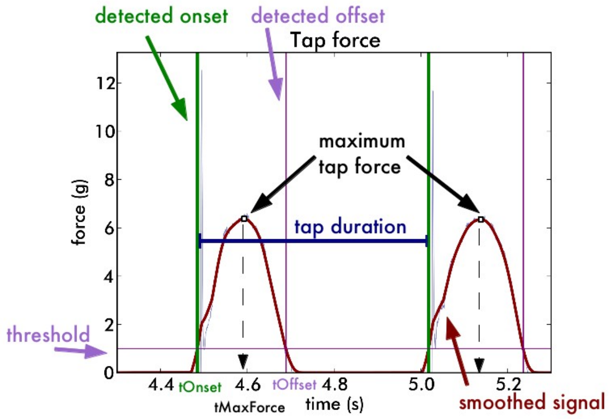

Supplement: Supplementary file 1 [file DataSheet1.PDF]
